# Prevalence and predictors of unintended pregnancy among women: an analysis of the Canadian Maternity Experiences Survey

**DOI:** 10.1186/s12884-015-0663-4

**Published:** 2015-10-13

**Authors:** Elizaveta Oulman, Theresa H. M. Kim, Khalid Yunis, Hala Tamim

**Affiliations:** School of Kinesiology and Health Science, York University, 4700 Keele Street, Toronto, Ontario M3J 1P3 Canada; Department of Pediatrics & Adolescent Medicine, American University of Beirut Medical Centre, Beirut, Lebanon

**Keywords:** Unintended pregnancy, Conception, Maternal health

## Abstract

**Background:**

Unintended pregnancies (mistimed or unwanted during the time of conception) can result in adverse outcomes both to the mother and to her newborn. Further research on identifying the characteristics of unintended pregnant women who are at risk is warranted. The present study aims to examine the prevalence and predictors of unintended pregnancy among Canadian women.

**Methods:**

The analysis was based on the 2006 Maternity Experiences Survey targeting women who were at least 15 years of age and who had a singleton live birth, between February 15, 2006 to May 15, 2006 in the Canadian provinces and November 1, 2005 to February 1, 2006 for women in the Canadian territories. The primary outcome was the mother’s pregnancy intention, where unintended pregnancy was defined as women who wanted to become pregnant later or not at all. Sociodemographic, maternal and pregnancy related variables were considered for a multivariable logistic regression.

**Results:**

Adjusted Odds Ratios (OR) and 95 % Confidence Intervals (95 % CI) were reported. Overall, the prevalence of unintended pregnancy among Canadian women was 27 %. The odds of experiencing an unintended pregnancy were statistically significantly increased if the mother was: under 20 years of age, immigrated to Canada, had an equivalent of a high school education or less, no partner, experienced violence or abuse and had 1 or more previous pregnancies. Additionally, mothers who reported smoking, drinking alcohol and using drugs prior to becoming pregnant, were all associated with an increased likelihood of experiencing an unintended pregnancy.

**Conclusion:**

The study findings constitute the basis for future research into these associations to aid in developing effective policy changes and interventions to minimize the odds of experiencing an unintended pregnancy and its associated consequences.

## Background

Unintended pregnancy is classified as pregnancies that are either mistimed or unwanted during the time of conception and can result in adverse outcomes both to the mother and to her newborn [1]. Based on these risks, women of childbearing age are recommended to practice preconception care in the form of adopting specific health-related behaviours [2]. However, many women with unintended pregnancies delay their prenatal care and engage in adverse health behaviours through the first trimester of their pregnancy [3–5]. For example, mothers with unintended pregnancies are more than twice as likely to report an inadequate consumption of folic acid prior to their pregnancy [3], putting their newborn at risk of developing neural tube defects [6, 7]. Studies have also shown that women with unintended pregnancies report a greater risk to alcohol exposure during the first trimester [5], exposing their newborns to elevated risks of developing abnormal fetal growth and morphogenesis [8]. Unintended pregnancies are adverse to the health of the mother as they put the mother at risk of developing mental health problems (*i.e.* depression) post partum [9, 10].

Knowledge on the dangers associated with unintended pregnancy is extensive, however more research is required on analyzing the characteristics of women who are at risk. Most of the research on the prevalence of unintended pregnancies is based on data from the United States. Studies report that the prevalence of unintended pregnancies ranges from one third upwards to one half of all births [3, 11, 12]. Research has found that the prevalence of unintended pregnancies was more than 50 % in women who were in age groups up to 24 years, living below the poverty line and/or had an education of less than 12 years [13]. Similarly, another study reports that the highest rates of unintended pregnancy tend to be among poorer women and those without a high school diploma [14]. Unfortunately, current data pertaining to unintended pregnancies among Canadian women is limited. The most recent crude estimate based on the 2006 Maternity Experiences Survey reports that approximately 27 % of Canadian mothers perceived their pregnancy to be as unintended, meaning that they would have preferred the pregnancy later or not at all [15].

Although the prevalence and dangers associated with unintended pregnancy has been examined in an array of studies [3–10, 13, 14] there is limited research among Canadian women. A nationwide study is needed in order to identify the characteristics and risk factors of Canadian women who experience an unintended pregnancy. Through the acquisition of this information, public health organizations will be better equipped at targeting interventions aimed at preventing unintended pregnancies among Canadian women and reducing the health risk for the mother and her newborn. Given the knowledge on the risks associated with unintended pregnancy and the scarcity of Canadian studies exploring unintended pregnancy, the present study aims at examining the factors associated with unintended pregnancy among Canadian women.

## Methods

### Study design

The current study was a cross-sectional design as the analysis was based on secondary data analysis. The database analyzed was the Maternity Experiences Survey.

### Database

This study is based on the secondary analysis of the 2006 Maternity Experiences Survey (MES), sponsored by the Canadian Perinatal Surveillance System of the Public Health Agency of Canada and conducted by Statistics Canada. The MES is the first and only Canadian survey devoted to pregnancy, labour, birth, and postpartum experiences; it has not been administered since 2006. The target population was selected from the 2006 Canadian Census of Population. This includes women who were at least 15 years of age at the time of birth, who had a singleton live birth in Canada and lived with their infants at the time of the survey. The birth cohort was selected from February 15, 2006 to May 15, 2006 for women living in the provinces and November 1, 2005 to February 1, 2006 for women living in the territories. Women excluded from the survey population included those who were under 15 years of age at the time of birth and mothers who lived in First Nations reserves or institutions during the time of the survey. Based on the above criteria, a total of 8542 women were eligible to participate in the survey, out of which 6421 women responded to the survey (response rate of 75.2 %). Data collection was obtained primarily through 45-min computer-assisted telephone interviews (except in the territories where in-person interviews were also utilized). Interviewers were trained on the purpose of the study and protocol for questionnaire administration. Interviews were conducted 5 to 10 months post-partum for women living in the provinces and 9 to 14 months post-partum for women living in the territories. The MES database has been presented to Health Canada’s Science Advisory Board, Health Canada’s Research Ethics Board and the Federal Privacy Commissioner and was approved by Statistics Canada’s Policy Committee [16]. Ethics approval was not necessary to obtain as this was based on secondary analysis of the MES collected by Statistics Canada. Access to the MES database was granted through the Research Data Centre in Toronto via an application submitted to the Social Sciences and Humanities Council of Canada. The design and methods of the MES has been previously described in other references [16].

### Outcome variable

The primary outcome of this study was the mother’s pregnancy intention. This variable was based on the question “Thinking back to just before you became pregnant, would you say that you wanted to be pregnant…?”. Response categories in the MES included the four categories: 1) sooner, 2) then, 3) later and 4) not at all. For the purposes of this study, pregnancy intention was assessed as a dichotomous variable where women who reported wanting to become pregnant “sooner/then” were coded as having an intended pregnancy and women wanting to become pregnant “later/not at all” were coded as having an unintended pregnancy.

### Independent variables

The potential predictors for unintended pregnancy included: 1) socio-demographic factors: age, place of residence (urban versus rural), immigration to Canada, aboriginal status and mother’s level of education; 2) maternal health characteristics: mother’s perceived health, previous depression diagnosis, pre-pregnancy body mass index (BMI), presence of partner or significant other and experience with violence over the last two years; and 3) pregnancy-related characteristics: number of past pregnancies, cigarette smoking before pregnancy, alcohol use before pregnancy and drug use before pregnancy. All of these variables, except experience with violence, were assessed using specific questions. Experience with violence was assessed over a set of ten questions about the mother’s experience with physical or sexual violence. A response of yes to any of the questions by the women was coded as having experienced violence or abuse.

### Statistical analysis

The prevalence of unintended pregnancy was estimated through survey weights created by Statistics Canada and provided with the MES data set. Differences in the predictors of unintended pregnancy were assessed at the bivariate level using normalized weights. Chi square tests were used to assess the association between the different levels of predictors and unintended pregnancy. Odds ratio (OR) with 95 % confidence intervals (95 % CI) were performed for all variables. To account for complex sampling design, bootstrapping was performed where appropriate to calculate all standard errors, the OR and 95 % CI estimates. The sample sizes reported in this manuscript were derived from normalized weights, weighted to represent a larger population. All analyses were computed with Stata Data Analysis and Statistical Software (version 13.0), and set at alpha <0.05 for two-tailed test for statistical significance.

## Results

**Fig. 1:**
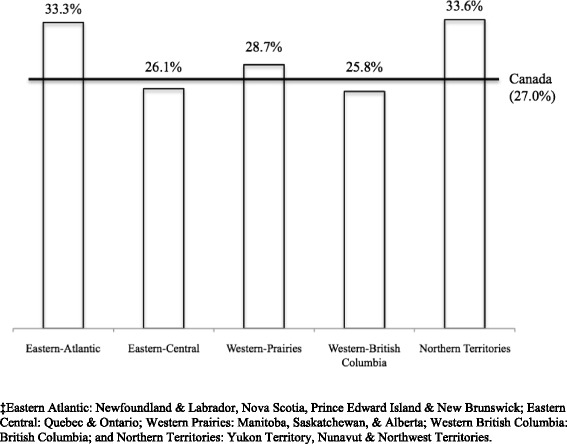
Distribution of women’s reaction to unintended pregnancy, across provinces and territories in Canada‡, 2006-2007

The sample size for the population analyzed in this study was 6421, weighted to represent 76,508 Canadian women. Out of 6421 women, a total of 6368 responded to the outcome variable “intended pregnancy” and were included in the analysis. As illustrated in Fig. 1, the prevalence of unintended pregnancy varied significantly (*p* < 0.05) across regions in Canada. Women in Eastern-Atlantic, Western-Prairie and the Northern Territories had the highest prevalence rates of unintended pregnancy; 33.3, 28.7 and 33.6 %, respectively. Women in the Eastern-Central provinces and Western-British Columbia reported prevalence rates of 26.1 and 25.8 %, respectively. These prevalence rates are below the reported Canadian prevalence of unintended pregnancy.

Table 1 presents the unadjusted and adjusted associations between unintended pregnancy and potential predictors. Analysis included a variety of maternal socio-demographic predictors such as age, urban-rural residence, aboriginal status, immigration to Canada, level of education and presence of a partner/ significant other. All of these predictors, with the exception of aboriginal status and residence in an urban-rural area were associated with unintended pregnancy in the adjusted model. Results demonstrate that women who were less than 20 years of age at the time of their pregnancy were more likely to experience an unintended pregnancy, compared to women who were 40 years of age and older (OR: 4.43; 95 % CI: 2.59, 7.58). In addition, women with an education equivalent to a high school diploma or less were 1.71 times more likely experience an unintended pregnancy compared to women who had graduate level education (95 % CI: 1.28, 2.29). Immigration to Canada and absence of a partner/ significant other were also found to be significant socio-demographic predictors of unintended pregnancy (OR = 1.53, 95 % CI: 1.27, 1.83; OR = 3.20, CI: 2.57, 3.99; respectively).

**Table 1:** Prevalence and predictors of unintended pregnancy based on a national survey of Canadian women (*N*=6,368)

	Sample Size	Unintended Pregnancy	Unadjusted Odds Ratio	Adjusted Odds Ratio
	N^a^	N (%)	OR (95% CI)^b^	OR (95% CI)^b^
Age (years)				
<20	187	72.6	*7.44 (4.75, 11.65)*	*4.43 (2.59, 7.58)*
20-29	2945	29.8	1.20 (0.84, 1.69)	1.19 (0.80, 1.76)
30-39	3018	21.7	0.78 (0.55, 1.11)	0.93 (0.63, 1.38)
≥40	185	25.9	1	1
Aboriginal Status				
Aboriginal	264	46.1	*2.41 (1.91, 3.04)*	1.29 (0.96, 1.73)
Non-Aboriginal	6074	26.2	1	1
Urban–rural residence				
Urban, population ≤499,999	2276	28.6	1.18 (1.00, 1.39)	1.14 (0.95, 1.37)
Urban, population ≥500,000	2780	26.2	1.05 (0.89, 1.23)	1.15 (0.96, 1.38)
Rural area	1090	25.3	1	1
Immigration to Canada				
Yes	1397	27.7	1.04 (0.89, 1.21)	*1.53 (1.27, 1.83)*
No	4942	26.9	1	1
Level of education				
High school or less	1316	41.3	*3.18 (2.49, 4.07)*	*1.71 (1.28, 2.29)*
Some postsecondary education	2748	26.7	*1.65 (1.30, 2.09)*	1.28 (0.99, 1.67)
Undergraduate education	1620	19.2	1.07 (0.83, 1.38)	1.05 (0.80, 1.37)
Graduate education	622	18.1	1	1
Partner/Significant other				
No	525	61.5	*5.06 (4.20, 6.11)*	*3.20 (2.57, 3.99)*
Yes	5818	23.9	1	1
Moms perceived health				
Poor/Fair	334	42.4	*2.29 (1.80, 2.90)*	*1.57 (1.18, 2.09)*
Good	1415	32.2	*1.48 (1.29, 1.69)*	*1.23 (1.05, 1.45)*
Excellent/very good	4615	24.4	1	1
Previous depression diagnosis				
Yes	980	33.1	*1.41 (1.21, 1.65)*	1.15 (0.97, 1.37)
No	5367	26.0	1	1
Pre-Pregnancy BMI (kg/m^2^)				
Underweight (<18.5)	380	33.9	1	1
Normal (≥18.5 & <25)	3700	25.8	*0.68 (0.54, 0.86)*	0.94 (0.72, 1.23)
Overweight (≥25 & <30)	1314	26.8	*0.72 (0.55, 0.93)*	0.98 (0.74, 1.32)
Obese (≥30)	850	29.7	0.83 (0.63, 1.08)	1.02 (0.75, 1.38)
Experienced violence within last 2 years				
Yes	689	44.6	*2.43 (2.07, 2.86)*	*1.34 (1.10, 1.63)*
No	5644	24.9	1	1
Number of past pregnancies				
1 or more	3453	27.6	1.06 (0.94, 1.19)	*1.33 (1.16, 1.52)*
None	2895	26.4	1	1
Cigarette smoking before pregnancy				
Yes	1399	40.0	*2.18 (1.92, 2.47)*	*1.34 (1.14, 1.57)*
No	4956	23.4	1	1
Alcohol use before pregnancy				
Yes	3964	27.5	1.06 (0.93, 1.20)	*1.17 (1.01, 1.35)*
No	2385	26.4	1	1
Drug use before pregnancy				
Yes	426	47.3	*2.61 (2.12, 3.21)*	*1.37 (1.05, 1.79)*
No	5927	25.6	1	1

^a^Sample size is estimated using normalized weights

^b^OR and 95% CI were calculated using bootstrapping technique

Maternal health characteristics considered in the analysis of this study included the mother’s perceived health, previous depression diagnosis, pre-pregnancy body mass index (BMI) and experience with violence within the last two years (at the time of the survey). Following adjustment, perceived health of the mother was observed as a strong predictor as women who reported their health as poor/fair or good had an increased likelihood of experiencing an unintended pregnancy compared to women who reported their health as excellent (OR = 1.57, 95 % CI: 1.18, 2.09; OR = 1.23, 95 % CI: 1.05, 1.45; respectively). Experience with violence remained significantly associated with unintended pregnancy through adjustment (OR = 1.34, 95 % CI: 1.10, 1.63). Although both the mother’s perceived health and experience with violence remained significant through adjustment, the associations were weaker in the adjusted model. Prior to adjustment, pre-pregnancy BMIs within the normal range (OR = 0.68, 95 % CI: 0.54, 0.86) and overweight range (OR = 0.72, 95 % CI: 0.55, 0.93) were found to have protective effects against unintended pregnancy when compared to women who reported themselves as underweight prior to pregnancy. However, this association lost its significance following adjustment. Previous depression diagnoses were not significantly associated with unintended pregnancy in the adjusted model.

Pregnancy-related characteristics such as number of past pregnancies, cigarette smoking, alcohol use and drug use before pregnancy were all found to be significant predictors of unintended pregnancy. Cigarette smoking and drug use before pregnancy were found to be the strongest predictors and remained significant through adjustment, although a weaker association was observed (OR = 1.34, 95 % CI: 1.14, 1.57; OR = 1.37, 95 % CI: 1.05, 1.79; respectively). Alcohol use before pregnancy was found to be a significant predictor after adjustment (OR = 1.17, 95 % CI: 1.01, 1.35). Interestingly, women who experienced 1 or more previous pregnancies were more likely to experience an unintended pregnancy compared to women who had no previous pregnancies in the adjusted model (OR = 1.33, 95 % CI: 1.16, 1.52).

## Discussion

The present study aimed to examine the potential predictors of unintended pregnancy among Canadian women. Pregnancies that are mistimed or unwanted can lead to adverse outcomes for both the mother and her newborn. Identifying the risk factors associated with unintended pregnancy can help with developing effective policy changes and interventions to minimize the odds of experiencing an unintended pregnancy and its associated consequences. The results of the present study indicate that the odds of experiencing an unintended pregnancy were increased if the mother was: under 20 years of age, immigrated to Canada, had an equivalent of a high school education or less, no partner, experienced violence or abuse and had one or more previous pregnancies. Additionally, mothers who reported smoking, drinking alcohol and using drugs prior to becoming pregnant, were all associated with an increased likelihood of experiencing an unintended pregnancy. Overall, the prevalence of unintended pregnancy across all Canadian provinces and territories was 27 %, with the highest prevalence in the Northern Territories and Eastern-Atlantic provinces, Newfoundland and Labrador.

The prevalence of unintended pregnancy in Canada is low when compared to the United States, which has a prevalence of approximately 51 % [12]. This difference can be attributed differences in healthcare system structure and population characteristics. With regard to maternal socio-demographics, mothers age, level of education, presence of a partner and immigration status were found to be significant predictors of unintended pregnancy following the multivariable analysis. The findings in the present study indicate that women who were less than 20 years of age at the time of their pregnancy were more likely to experience their pregnancy as unintended, compared to those who were over 40 years of age. These findings are consistent with those in the existing literature [17–19]. However, earlier research findings from the United States has found the opposite, suggesting that women who were over 35 years of age were more likely to experience an unintended pregnancy as they may have already had all the children they wanted to have [13]. The findings from this study should be considered with caution, as this data is older than the literature previously listed [17–19], suggesting that this data may be outdated. In comparison to women with graduate level education, women with an education level equivalent to a high school diploma or less were found to be at a higher risk of reporting an unintended pregnancy, consistent with earlier research [13, 14, 18, 20]. To support these findings, it has been suggested that women with a high school education (or less) may perceive a pregnancy as jeopardizing any potential career plans or aspirations [13]. The findings from the present study also demonstrate that women who do not have a partner or significant other, were 3.20 (95 % CI: 2.57, 3.99) times more likely to experience an unintended pregnancy compared to those who have a partner or significant other. Although the presence of a partner or significant other does not imply that the woman is married, existing literature has shown that unmarried or single women, compared to those who are married, were more likely to experience an unintended pregnancy [19, 20]. Results also indicated that immigrant women were more likely to experience an unintended pregnancy however, no previous studies have examined the association of this variable with unintended pregnancy. Women who are immigrants may lack the financial stability and social support to have a newborn child. The significance of these findings suggests that future research into this area should be considered.

The present study found that experience with violence within the last two years of the pregnancy and the mothers perceived health were significant variables associated with experiencing an unintended pregnancy. These findings are consistent with previous research, which indicates that women are more likely to perceive their pregnancy as unintended if they have experienced violence or abuse from their partner [21–23]. Experiences with violence and/or abuse have been found to foster environments of fear and a loss of control in relationships, leading to perceptions of a new pregnancy as unintended [24]. The results also found that women who reported their health as poor/fair or good, compared with those who reported their health as excellent, were more likely to experience an unintended pregnancy. Support in the literature is sparse however, one study has found that women with poorer physical and mental health status were more likely to experience an unintended pregnancy [25]. Childbearing can require a high level of physical and emotional stability, thus mothers who perceive themselves as having poor health may be more likely to report a new pregnancy as unintended.

At the multivariable level, all pregnancy related characteristics were found to be significant predictors of unintended pregnancy. The results indicate that women with previous pregnancies were more likely to report an unintended pregnancy compared to those who are nulliparous. These findings are supported by the existing literature [13, 18–20]. Similar to women over 35 years of age, it can be suggested that women who have had previous children may already have all of the children they want, thus, reporting any new pregnancies as unintended [13]. Additionally, engaging in behaviours such as smoking, alcohol and drug use prior to pregnancy were all associated with an increased odds of experiencing an unintended pregnancy, compared to women who did not engage in these behaviours. There is consistent literature highlighting the relationships between smoking [3, 18, 26], alcohol [5, 26] and drug use [18, 26] with unintended pregnancy. Considering the dangers associated with these behaviours and pregnancy [27], women who engage in these behaviours prior to becoming pregnant may not have any intentions of becoming pregnant at the time or later, resulting in the experience of a new pregnancy as unintended.

The results of the present study should be interpreted with care as limitations are imposed. A major limitation of the current study is that all data was collected through self-report measures, increasing the possibility of recall bias. Additionally, potential for misclassification bias of the outcome “unintended pregnancy” exist due to the framing of the question; as it implies either unplanned pregnancy (*i.e.*, related to timing issues) or unwanted pregnancy (*i.e.*, not desired, related to the situation or persons involved). The cross-sectional design of the current study is also limiting, as it does not allow causality to be inferred. Although the MES database was surveyed back in 2006, it is the first and only Canadian survey devoted to pregnancy and maternal experiences that is representative at the national level across all provinces and territories, with a response rate of 75.2 %. Furthermore, the present study considered a variety of predictors across various domains, mitigating the effects of any confounding factors. Despite the limitations, this study serves as an important baseline that could be used to compare to other countries and provide as a lead for future research considerations in the area of unintended pregnancy.

## Conclusion

The present study has identified an important public health priority in the area of maternal health and pregnancy, as unintended pregnancy is associated with detrimental effects on both the mother and her newborn. In Canada, approximately 27 % of all pregnancies are reported as unintended by the mother, meaning that the mother wanted to become pregnant later or not at all. Results of the current study identified predictors of unintended pregnancy in Canadian women and provide the basis for future research into these associations. Furthermore, our findings may benefit public health organizations in the area of unintended pregnancy as they can be used as the basis for designing effective interventions to decrease the risk of unintended pregnancy, specifically focusing on mothers who are young, single and low educated. Finally, educating mothers on the dangers and risk factors associated with unintended pregnancy is warranted.

## Abbreviations

BMI: Body mass index
CI: Confidence interval
MES: Maternity experiences survey
OR: Odds ratio
